# Incidence of suspicious axillary lymph node involvement in fluorine-18 fluoro-D-glucose positron emission tomography/computed tomography in gynecologic cancers

**DOI:** 10.4274/tjod.12144

**Published:** 2018-06-21

**Authors:** Jalal Raoufi, Serhan Can İşcan, Candost Hanedan, Emine Elif Özkan, Sevim Süreyya Çerçi, Ebru Erdemoğlu, Evrim Erdemoğlu

**Affiliations:** 1 Süleyman Demirel University Faculty of Medicine, Department of Obstetrics and Gynecology, Division of Gynecologic Oncology, Isparta, Turkey; 2 Süleyman Demirel University Faculty of Medicine, Department of Radiation Oncology, Isparta, Turkey; 3 Süleyman Demirel University Faculty of Medicine, Department of Nuclear Medicine, Isparta, Turkey; 4 Isparta Maternity and Children’s Diseases Hospital, Clinic of Obstetrics and Gynecology, Isparta, Turkey

**Keywords:** Gynecologic cancers, maximum standardized uptake value, positron emission tomography/computed tomography, metachronous, axillary lymph node

## Abstract

**Objective:**

There is scarce information about axillary lymph node involvement in gynecologic cancers. We analyzed the incidence of suspicious axillary lymph nodes in gynecologic cancers.

**Materials and Methods:**

We retrospectively analyzed the positron emission tomography/computed tomography findings of 251 patients with endometrial, cervical, and ovarian cancer. There is no cut-off value documented for axillary metastases from gynecologic cancers; therefore we adopted the cut-off standardized uptake values (SUVs) proclaimed in breast cancer.

**Results:**

A total of 251 patients records were available for analysis; 40 patients (15.9%) with suspicious axillary lymph nodes were included in the study. Twenty-one and a half percent (n=20/93) of patients with endometrium cancer, 14.1% (n=14/99) of patients with ovarian cancer, and 10% (n=6/59) of those with cervical cancer had suspicious axillary lymph nodes. Patients with an maximum SUV (SUV_max_) uptake higher than 3 underwent axillary lymph node biopsy. None of them was found to have axillary metastases of gynecologic cancers in the pathologic evaluation. In one patient with endometrial cancer, an obscure breast ductal carcinoma was diagnosed, another patient with endometrial cancer was found to have follicular lymphoma. The third patient with endometrial cancer had no malignancy in axillary lymph node biopsy, but had Hurthle cell neoplasia in a thyroid biopsy; the patient did not accept any surgical or medical treatment for endometrial cancer and died 23 months later. There were three (7.5%) metachronous cancers out of 40 gynecologic cancers; two patients were explained above, the third patient with endometrium cancer, who was not histopathologically evaluated although the axillary SUV_max_ was <3, had rectosigmoid cancer and glioblastoma metachronously.

**Conclusion:**

Our study shows that an important ratio (14-21%) of patients with gynecologic cancer has suspicious axillary lymph nodes. Increased SUV_max_, particularly above 3, might be used as an indication for axillary biopsy and may help to identify secondary metastatic cancer.

**PRECIS:** In gynecologic cancers, patients with suspicious axillary lymph nodes need to be evaluated and further investigated to exclude other causes.

## Introduction

Two-deoxy-2-[fluorine-18] fluoro-D-glucose (^18^F-FDG) positron emission tomography (PET) adds valuable data based on the increased glucose uptake and depicts metabolic abnormalities before morphologic alterations occur. PET has been widely used in staging, pre-operative planning, and follow-up of gynecologic cancers in our department, as well as worldwide. Whole-body acquisition by ^18^F-FDG PET/computed tomography (CT) imaging may demonstrate unusual findings in distant unexpected localizations^(1)^. Patients with a gynecologic malignancy are at greater risk of developing synchronous or metachronous secondary cancers, therefore these unusual ^18^F-FDG PET/CT findings may be important. Besides, skip metastases to the axillary lymph nodes may influence surgical and adjuvant treatment of the patient. We have noticed that in an important part of patients with gynecologic cancer, ^18^F-FDG PET/CT has indicated incidental axillary lymph nodes although axillary lymphadenopathy is rare in gynecologic cancers^(1)^. There is a paucity of information about axillary lymph node involvement in gynecologic cancers in terms of prognostic importance and accurate management. The aim of the present study was to analyze the incidence of suspicious axillary lymph nodes in gynecologic cancers and evaluate the oncologic and ^18^F-FDG PET/CT features.

## Materials and Methods

We retrospectively analyzed the ^18^F-FDG PET/CT findings of 251 patients with endometrial, cervical, and ovarian cancer who were referred to our clinic between 2010 and 2017. We included gynecologic oncology patients with suspicious axillary lymph nodes when they had one of the following features in ^18^F-FDG PET/CT imaging: lymph nodes with a diameter equal to or larger than 10 mm, parenchymal thickening and loss of fatty hilum, or increased FDG-uptake value equal to or greater than 1.8. There is no cut-off value documented for axillary metastases from gynecologic tumors; therefore, we adopted the cut-off standardized uptake value (SUV) proclaimed in breast cancer. However, there is also no consensus for SUV values for the detection of axillary metastases in breast cancer. An SUV cut-off value of axillary metastases in breast cancer set at 1.8 or more is reported to have 35.6% sensitivity, 100% specificity, and 100% positive predictive value^(2)^. In other studies, the optimal cut-off level of maximum SUV (SUV_max_) on PET/CT for malignant isolated axillary lymph nodes was reported as 3.01^(3)^. Women with an SUV uptake higher than 3 underwent histopathologic evaluation through fine-needle core biopsy, excisional biopsy or complete axillary lymph node dissection according to the surgeon’s preference. Demographic and oncologic characteristics of the patients, ^18^F-FDG PET/CT findings, SUV_max_, follow-up of axillary lesions, and biopsy results were recorded. FDG-PET image acquisition and whole-body FDG-PET scans were performed as described using the Philips Gemini TF PET/CT scanner (Philips Medical Systems B.V., Eindhoven, The Netherlands). Patients were prepared with a 6 h fast because serum glucose levels had to be <150 mg/dL prior to glucose tracer administration. At 60 min after the intravenous injection of 3.7 MBq/kg (0.1 mCi/kg) ^18^F-FDG (Monrol, Eczacıbaşı, İstanbul, Turkey), PET/CT was performed. Subsequently, an emission scan was recorded in the three-dimensional mode following CT for 2 min per position. PET and CT images were examined in the cross-sectional planes view and in the rotating maximum-intensity projection. The study was approved by the Süleyman Demirel University Local Ethics Committee (approval number: 175 dated 04.10.2017).

### Statistical Analysis

Statistical analyses were performed using the MedCalc Software (version 17.4.4, Belgium) and IBM SPSS Statistics 24 Software. One-way ANOVA and the chi-square test were used to compare variables. A p value of 0.05 or less was defined as statistical significance.

## Results

**Figure 1:**
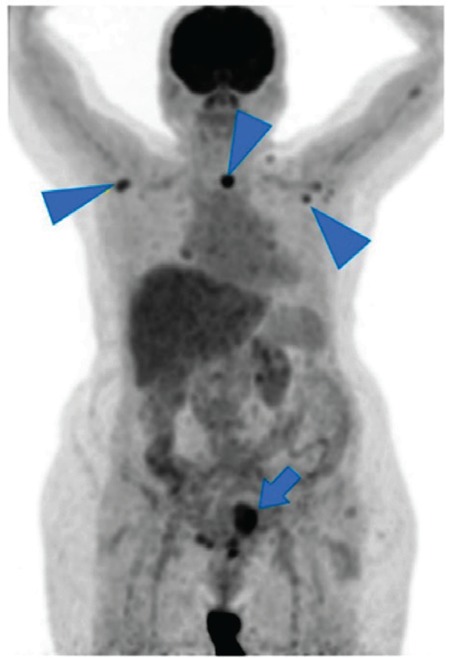
Positron emission tomography/computed tomography maximum intensity projection image shows increased fluoro-D-glucose uptake in uterine cavity (arrow), bilateral axillary lymph nodes and nodular lesion in left thyroid lobe (arrowhead)

**Figure 2:**
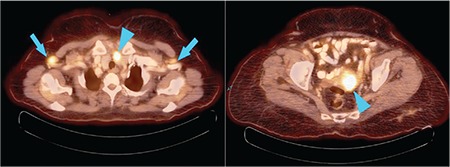
a) Positron emission tomography/computed tomography fusion image of the thorax. The image shows bilateral axillary hypermetabolic axillary lymph nodes [maximum standardized uptake value (SUV_max_): 4.72] (arrow), a hypermetobolic nodular lesion in the left lobe of the thyroid was found incidentally (SUV_max_: 7.72) (arrowhead). Fine-needle aspiration of thyroid revealed Hurthle cell neoplasia, b) Hypermetabolic mass in the uterus with a SUV_max_ value of 9.42 (arrowhead)

A total of 251 patient records were available for analyses. Forty (15.9%) patients with suspicious axillary lymph node metastases were included in the study. The mean age of patients was 60.4±9.1 years. Axillary lymph node involvement was most commonly observed in patients with endometrium cancer. There were 93 patients with endometrium cancer and a suspicious lymph node was found in 21.5% (n=20). There were 99 patients with ovarian cancer and 14.1% (n=14) had a suspicious axillary lymph node in ^18^F-FDG PET/CT imaging. Ten percent (n=6/59) of patients with cervical cancer had suspicious axillary lymph nodes (Table 1). In 17 patients (out of 40), suspicious axillary findings were evident in pre-operative/treatment PET/CT. Twenty-three patients (out of 40) had no suspicious axillary lymph nodes in preoperative/treatment PET/CT; suspicious axillary lymph nodes were found as a new findings in the follow-up of these patients (Table 2). In follow-up of the 40 patients with suspicious axillary lymph nodes, 28 (70%) patients had regression, 3 (7.5%) progressed, and 9 (22.5%) patients remained stable. Of the three progressive cases, one patient with endometrial cancer did not accept any surgical or medical treatment for endometrial cancer and died 23 months after ^18^F-FDG PET/CT imaging. The second patient had stage IIIB ovarian cancer and received 6 courses of carboplatin-paclitaxel chemotherapy (3 neoadjuvant and 3 adjuvant); the suspicious lymph node regressed after chemotherapy. The third patient with progressive suspicious axillary lymph node had stage IIIC ovarian cancer, she received 3 courses of carboplatin + paclitaxel chemotherapy before surgery, and was followed up with 36 courses of chemotherapy after surgery, (6 courses of carboplatin + paclitaxel, 6 courses of bevacizumab, 6 courses of carboplatin + paclitaxel, 6 courses of gemcitabine, 6 courses of topotecan, 6 courses of doxorubicin); however, despite this intense chemotherapy, the disease progressed and she died after 42 months’ survival. The treatment and follow-up of 40 patients with the suspicious axillary lymph node in PET-CT is shown in Tables 3 to 5. In our last follow-up, 18 patients out of the 40 had died, 16 of which of their cancer, and 2 of other causes (Table 4). Eighteen of the remaining 22 patients were in remission, 3 patients had progression, and 1 patient was lost to follow-up. Twenty-one of the 40 patients had no FDG uptake but had obviously enlarged axillary lymphadenopathy in ^18^F-FDG PET/CT imaging, and 19 patients had FDG uptake in the axilla (Table 1, Figure 1, 2). The mean SUV_max_ in axillary lymph nodes with FDG uptake (SUV_max_) was 2.4±2.3. Forty-two percent of patients had an SUV_max_ higher than the cut-off value of 1.8 (Table 2). Patients with an SUV_max_ uptake higher than 3 underwent axillary lymph node biopsy. There were 6 patients with an SUV_max_ >3. All but one gave informed consent for an intervention for histopathologic verification. None of the patients was found to have axillary metastases in the pathologic evaluation. In one patient with endometrial cancer, an obscure breast ductal carcinoma was diagnosed, the patient had stage IIIC breast cancer with axillary metastasis. She underwent surgical treatment for breast cancer along with surgical treatment for endometrial cancer. She received adjuvant chemo-radiotherapy for breast cancer and was disease-free in her last follow-up. The second patient was found to have follicular lymphoma and sent to hematology after surgical treatment of endometrial cancer. The third patient with endometrial cancer had no malignancy in her axillary lymph node biopsy, but had Hurthle cell neoplasia in a thyroid biopsy; she refused surgical or medical treatment for endometrial cancer and died 23 months later. The other patients who underwent axillary lymph node biopsy had no malignancy at the pathologic evaluation. In the sixth patient with cervical cancer; initially, there was a suspicious lymph node with an SUV_max_ equal 3.64, which was interpreted as reactive, but it could not be observed in the second ^18^F-FDG PET/CT imaging after the first-line chemo-radiotherapy before the biopsy. In the last follow-up, she was in remission. The features of patients who underwent axillary biopsy are shown in Table 5.

## Discussion

We found that an important proportion of gynecologic oncology patients had suspicious lymphadenopathy in ^18^F-FDG PET/CT imaging, in particular, a significant proportion of patients with endometrium and ovarian cancer (21.5% and 14.1%, respectively). In our study, 52.5% of these patients had enlarged axillary lymph nodes without FDG uptake and 47.5% of these patients had enlarged axillary lymph nodes with high SUV uptake. The mean axillary SUV_max_ was 2.4±2.3. PET–CT is reported to have low sensitivity but high specificity in breast cancer, which indicates that it is more useful when metastasis is suspected. There is currently no consensus regarding the differentiation of benign from malignant lymph nodes and there is also no agreement on the cut-off for SUV_max_ values^(4)^. Axillary lymph node metastases are usually expected in primary tumors of the breast, lung, thyroid, stomach, skin, and ovary^(5)^. The most common source of axillary lymph node metastasis is breast cancer, metastasis from non-mammary primary cancer to the axillary lymph nodes is less than 3%^(6)^. Metastases from a gynecologic malignancy are considered extraordinary. Axillary lymph node metastases in endometrium cancer are reported as 0.03% by Aalders et al.^(7)^. Axillary involvement in ovarian cancer has been reported as case series and is usually associated with breast metastases^(8)^. Serous histology in ovarian cancer was the most important risk factor for axillary metastasis. Euscher et al.^(9)^ studied 35 patients with ovarian, fallopian tube or peritoneal serous carcinoma that presented as lymphadenopathy. In their study, there were only 2 axillary lymphadenopathies with peritoneum primary sites^(9)^. Sangle et al.^(10)^ reported a 63-year-old patient who had serous carcinoma of the fallopian tube with axillary lymph node involvement. Isolated axillary lymph node metastasis from serous ovarian cancer has also been published as case reports in the literature^(11,12)^. Skagias et al.^(8)^ reported a patient with ovarian carcinoma that presented with axillary lymph node metastasis. Sanuki et al.^(5)^ reported a 57-year-old woman with endometrium cancer with axillary lymph node involvement in Japan in 2007. As a determination of their study to evaluate a cut-off value regarding the differentiation of benign from malignant lymph nodes, Kyoung^(3)^ reported that axillary lymph nodes with isolated FDG uptakes in whole-body PET/CT showed a low risk of malignancy (25%), and axillary lymph nodes with an SUV greater than 3.01 were malignant. Therefore, in our protocol, we suggested histopathologic evaluation of axillary lymph nodes in patients with an SUV_max_ >3. In our study, 6 patients had an SUV_max_ higher than 3. All but one gave informed consent for an intervention for histopathological verification. However, management and follow-up for axillary lymph nodes with abnormal visual and functional findings in PET/CT in patients with endometrial or ovarian cancer are not consistently established. It is uncommon to detect a synchronous or metachronous secondary cancer in reproductive system malignancies. The incidence of occult breast cancer is reported as 0.3-1% of all patients with breast cancer. Occult breast cancer is the most likely diagnosis associated with axillary metastasis^(13)^. Sughayer et al.^(14)^ reported a 63-year-old woman with collision axillary metastasis from breast cancer and ovarian cancer. Atallah et al.^(6)^ reported a case of ipsilateral breast cancer and occult tubal serous carcinoma. In our study, there were 3 (7.5%) metachronous cancers out of 40 gynecologic cancers with suspicious axillary lymph nodes in PET/CT imaging, which is reported as uncommon in previous literature. The first patient with endometrial cancer, an obscure breast ductal carcinoma, was diagnosed through axillary lymph node biopsy, she had stage IIIC breast cancer with axillary metastasis. The second patient was found to have follicular lymphoma via axillary lymph node biopsy and was sent to hematology after surgical treatment for endometrial cancer. The third patient, who had stage IB endometrial cancer, was not histopathologically evaluated because of having an axillary SUV_max_ <3, had rectosigmoid cancer and glioblastoma metachronously. This patient died of glioblastoma. This high metachronous cancer ratio warrants detailed evaluation of patients with gynecologic cancer with axillary lymph node abnormalities in ^18^F-FDG PET/CT for secondary primary cancer.

## Conclusion

Our study shows that an important ratio (14-21%) of patients with gynecologic cancer has suspicious axillary lymph nodes. Increased SUV_max_, particularly higher than 3, might be used as an indication for axillary lymph node biopsy and may help to identify secondary metastatic cancer. Axillary metastases in gynecologic cancers may up-stage the patient to stage IV and may preclude aggressive cytoreductive surgery or may ensure adjuvant chemotherapy. Patients with increased SUV uptake higher than 3 in ^18^F-FDG PET/CT imaging need to be evaluated, and further investigations should be performed to exclude other causes of lymphadenopathy.

## Figures and Tables

**Table 1:**
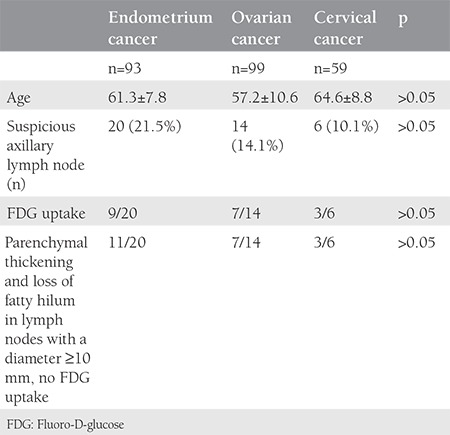
Characteristic of gynecologic oncology patients with suspicious axillary imaging in positron emission tomography/computed tomography

**Table 2:**
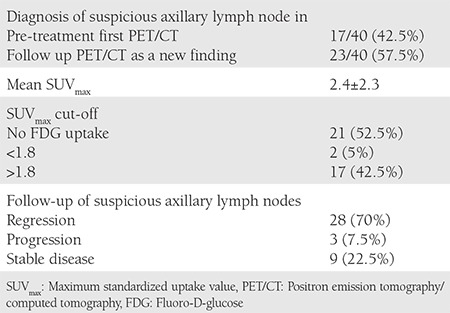
Positron emission tomography/computed tomography features, maximum standardized uptake value values, and results of a suspicious axillary lymph node imaging

**Table 3:**
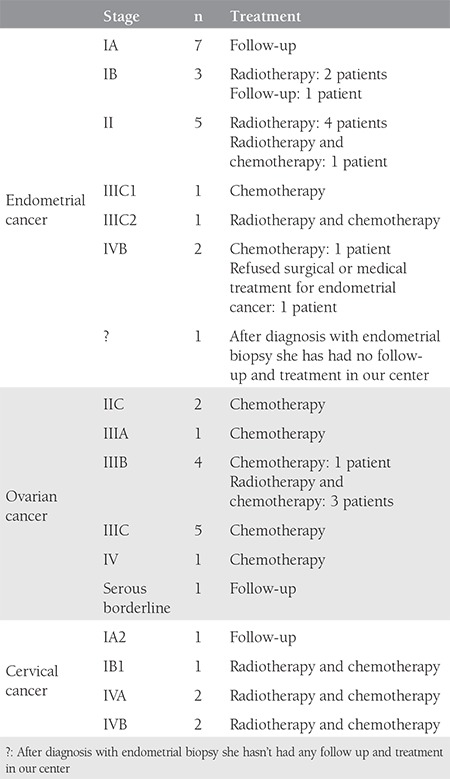
The treatment and follow-up of 40 patients with suspicious axillary lymph node in positron emission tomography/computed tomography

**Table 4:**
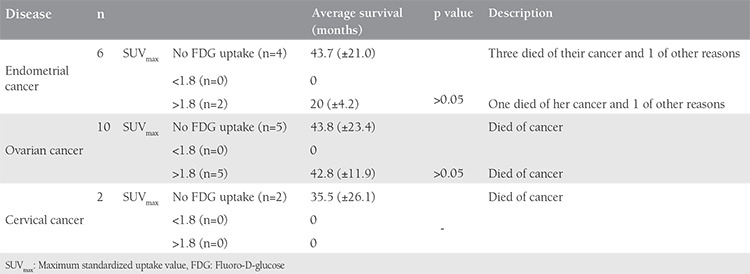
Deceased patients according to diagnostic subtype

**Table 5:**
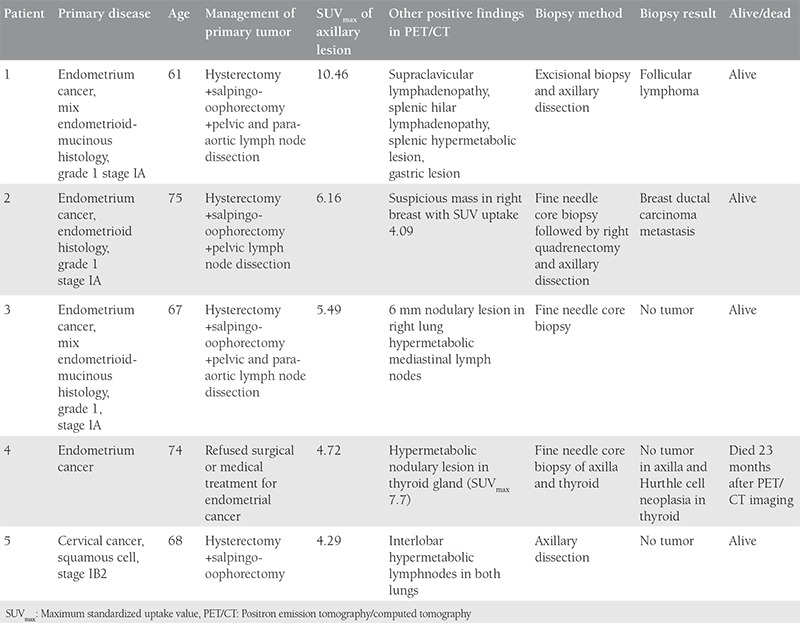
Patients who had axillary lymph node standardized uptake value uptake >3 had intervention for histopathologic evaluation. Features of patients who had an axillary needle core biopsy. No patient was found to have axillary metastases in the pathologic evaluation
